# Emergence of a NDM-1-producing ST25 *Klebsiella pneumoniae* strain causing neonatal sepsis in China

**DOI:** 10.3389/fmicb.2022.980191

**Published:** 2022-10-20

**Authors:** Junhui Zhao, Beiwen Zheng, Hao Xu, Junfeng Li, Tengfei Sun, Xiawei Jiang, Wenhong Liu

**Affiliations:** ^1^School of Basic Medical Sciences, Zhejiang Chinese Medical University, Hangzhou, China; ^2^Collaborative Innovation Center for Diagnosis and Treatment of Infectious Diseases, State Key Laboratory for Diagnosis and Treatment of Infectious Diseases, The First Affiliated Hospital, College of Medicine, Zhejiang University, Hangzhou, China

**Keywords:** CRKP, *bla*_NDM-1_, ST25, neonatal sepsis, WGS

## Abstract

Carbapenem-resistant *Klebsiella pneumoniae* (CRKP) seriously threaten the efficacy of modern medicine with a high associated mortality rate and unprecedented transmission rate. In this study, we isolated a clinical *K. pneumoniae* strain DY1928 harboring *bla*_NDM-1_ from a neonate with blood infection. Antimicrobial susceptibility testing indicated that DY1928 was resistant to various antimicrobial agents, including meropenem, imipenem, ceftriaxone, cefotaxime, ceftazidime, cefepime, piperacillin-tazobactam, and amoxicillin-clavulanate. S1 nuclease-pulsed field gel electrophoresis (S1-PFGE), southern blot and conjugation experiment revealed that the *bla*_NDM-1_ gene was located on a conjugative plasmid of IncA/C2 type with a 147.9 kb length. Whole-genome sequencing showed that there was a conservative structure sequence (*bla*_NDM-1_-*ble*-*trpF*-*dsbD*) located downstream of the *bla*_NDM-1_ gene. Multilocus sequence typing (MLST) classified DY1928 as ST25, which was a hypervirulent *K. pneumoniae* type. Phylogenetic analysis of genomic data from all ST25 *K. pneumoniae* strains available in the NCBI database suggested that all *bla*_NDM-1_ positive strains were isolated in China and had clinical origins. A mouse bloodstream infection model was constructed to test the virulence of DY1928, and 11 *K. pneumoniae* strains homologous to DY1928 were isolated from the feces of infected mice. Moreover, we found that DY1928 had a tendency to flow from the blood into the intestine in mice and caused multiple organ damage. To our knowledge, this is the first study to report an infection caused by *bla*_NDM-1_-positive ST25 *K. pneumoniae* in the neonatal unit. Our findings indicated that stricter surveillance and more effective actions were needed to reduce the risk of disseminating such *K. pneumoniae* strains in clinical settings, especially in neonatal wards.

## Introduction

Sepsis is one of the three most common causes of neonatal deaths globally (Denis, 2016). It was found that about 40% of newborns die from severe neonatal infections by a multi-country prospective cohort study in Asia (Amanhi, 2018). In this situation, carbapenem antimicrobials have become the last line of defense in the treatment of neonatal infection due to its broad antibacterial spectrum (Akhtar et al., 2020). An increasing number of carbapenem-resistant *K. pneumoniae* (CRKP) infections have rapidly spread worldwide due to the extensive use of carbapenem antimicrobials, and their morbidity and mortality have significantly increased (Agyeman et al., 2020). In recent years, outbreaks of neonatal CRKP infections have been reported and have attracted widespread attention (Datta et al., 2017; Huang et al., 2018).

New Delhi metallo-β-lactamase (NDM) is a metallo-β-lactamase capable of hydrolyzing most β-lactams, including carbapenems. Therefore, the bacteria carrying this enzyme are called “superbugs” (Wu et al., 2019). Among the NDM-producing Enterobacteriaceae, *K. pneumoniae* is the predominant carrier (Wu et al., 2019). NDM was first identified in a *K. pneumoniae* strain isolated from patients hospitalized in New Delhi, India, in 2008 (Yong et al., 2009). Since then, 33 variants of NDM have been described (Wang et al., 2021). As treatment selection is limited to a very small number of antimicrobials, such as colistin and tigecycline, hospital-acquired and community-acquired infections caused by NDM-producing bacteria are difficult to eliminate (Guducuoglu et al., 2018). Since the first report of *K. pneumonia* carrying *bla*_NDM-1_ in Nanchang, 2013, this superbug has spread rapidly throughout China (Huang et al., 2018). CRKP have been classified as a critical priority superbug by the World Health Organization (WHO), indicating new treatment and prevention strategies are urgently needed. Therefore, studies on the surveillance and antimicrobial resistance patterns of CRKP are of critical importance (Wyres et al., 2020).

Several studies have reported that ST25 *K. pneumoniae* caused hospital-acquired infections (Cienfuegos-Gallet et al., 2019). It was also the main sequence type of carbapenem-resistant hypervirulent *K. pneumoniae* (CR-hvKP) strains in the hospital in mid-south China (Li et al., 2019). However, to our knowledge, there is no report of neonatal sepsis caused by ST25 *K. pneumoniae*. The most commonly reported ST types of *K. pneumoniae* that caused neonatal sepsis in China were ST105, followed by ST17 and ST20 (Ding et al., 2019). In contrast, information on the virulence of ST25-type CRKP in neonates remains elusive. In the present study, we characterized the phenotype, genotype and virulence of a clinical ST25 *K. pneumoniae* strain DY1928 harboring *bla*_NDM-1_ isolated from a neonate with sepsis for the first time. Furthermore, we explored the resistance mechanism of DY1928, characterized the genetic environment and delivery pattern of the plasmid carrying *bla*_NDM-1_ gene from DY1928, and revealed its phylogenetic features.

## Materials and methods

### Ethics statement

All animal assays were approved by the Ethics Committee of Zhejiang Chinese Medical University (Hangzhou, Zhejiang Province, China). All the participants provided consent prior to the study. Patient involved in the study was anonymized, and no informed consent was acquired because of the retrospective study.

### Clinical information of the patient

A 9-day-old female neonate who was born prematurely underwent cerebrospinal fluid (CSF) routine and blood culture due to secondary infection in October 2019. Based on the findings from the CSF and other indicators, the neonate was diagnosed with purulent meningitis. The initial blood culture report indicated that the neonate had a nosocomial infection with gram-negative bacteria. Because this type of infections often involve bacteria that are resistant to beta-lactam antibiotics, the neonate was treated empirically with a combination of penicillin sodium and meropenem. The tertiary level report of blood culture showed that the infection was caused by CRKP. The treatment was successful, as shown by the blood culture, which showed negative for two consecutive times, and the CSF routine was normal. The neonate gradually improved and finally recovered.

### Bacteria isolation and identification

Blood culture was initially performed in the hospital clinical laboratory. Briefly, blood samples were inoculated into aerobic and anaerobic blood culture bottles and then incubated in the in BACTEC blood culture system (BD Diagnostics, United States). Once reported positive, a gram stain was performed from the bottle and the broth was plated onto 5% sheep blood agar and MacConkey agar. The plates were incubated at 37°C and then inspected for growth at 24 h and 48 h. The bacterial isolate was first identified by matrix-assisted laser desorption/ionization time-of-flight mass spectrometry (MALDI-TOF/MS) (Bruker Daltonik GmbH, Bremen, Germany). Antimicrobial susceptibility testing was performed using the Kirby-Bauer disc diffusion method and Vitek 2. A single clone of the isolate from blood culture was picked and inoculated onto a blood plate for gorwth at 37°C for 24 h. Then the purified strain was stored in 20% glycerol cryogenic vials at −80°C.

### Detection of *bla*_NDM_

To detect the presence of *bla*_NDM_, polymerase chain reaction (PCR) and agarose gel electrophoresis were performed, and a known *bla*_NDM_-positive control (previously characterized strain) was included. The self-designed and synthesized, universal primers used here were as follows: *bla*_NDM_ forward, 5’-ATGGAATTGCCCAATATTATGCAC-3′; *bla*_NDM_ reverse, 5’-TCAGCGCAGCTTGTCGGC-3′. PCR was performed in a total reaction volume of 50 μl containing 21 μl of Taq polymerase Mix (Pharmacia), 1 μl of each primer (10 μmol/l), 2 μl of template DNA, and 25 μl of nuclease-free water. With 30 cycles of amplification (45 s at 95°C, 30 s at 50°C, and 45 s at 72°C), the amplified products were visualized after 15 min of electrophoresis on a 1% agarose gel containing ethidium bromide.

### Whole-genome sequencing

Bacterial genomic DNA was extracted using a Qiagen DNA purification kit (QIAGEN, Hilden, Germany), and the quality of the extracted DNA was evaluated using a Nanodrop™ spectrophotometer (Thermo Fisher Scientific, Massachusetts, United States) (Zheng et al., 2014). Then the genomic DNA was sent to Novogene (Beijing Novogene Bioinformatics Co., Ltd., Beijing, China) for WGS using Illumina HiSeq 4,000 (Illumina, San Diego, CA, United States) combined with the Oxford Nanopore MinION platform (Nanopore Technologies, Oxford, United Kingdom) (Xiaoliang et al., 2019). The sequencing process was up to 48 h long, and raw fast 5 files were generated using the MinKNOW software (Vijayakumar et al., 2020). *De novo* assembly was performed using Unicycler (Wick et al., 2017) and SPAdes genome assembler (Prjibelski et al., 2020). Finally, a complete and accurate genome assembly was generated by Pilon (Walker et al., 2014), and the quality, completeness and contiguity of it were verified using QUAST (Gurevich et al., 2013). The whole-genome sequence of DY1928 was deposited in GenBank^1^ under the following accession numbers: CP090429 for the chromosome and CP090430 for the plasmid.

### Genome annotation

The antimicrobial resistance genes (ARGs) of DY1928 were predicted using ResFinder 4.1,^2^ and the plasmid was typed using PlasmidFinder 2.1.^3^ The genome of DY1928 was annotated by the RAST^4^ server. The insertion sequence (IS) and transposons were identified using the ISFinder^5^ database. The virulence factors were annotated by blasting the VFDB^6^ database. Multiple plasmid comparison was generated using the BLAST Ring Image Generator (BRIG).^7^ The genetic environment surrounding the *bla*_NDM-1_ gene was visualized by Easyfig.^8^ The sequence type (ST) was detected by the online database MLST 2.0^9^ based on sequence analysis of loci from 7 housekeeping genes: *gapA*, *infB*, *mdh, pgi*, *phoE*, *rpoB*, and *tonB*. Moreover, the CRISPR-Cas system was characterized by CRISPRCasFinder,^10^ and the prophage was identified by PHAST^11^ server.

### Phylogenetic tree construction

The whole-genome sequences of 90 ST25 *K. pneumoniae* strains were downloaded from the NCBI^1^ database. We uploaded sequence files of all these strains and DY1928 to the Prokka^12^ server for annotation. The annotation files were then uploaded to Roary^13^ for alignment. MEGA11^14^ was used to construct a maximum likelihood tree through the alignment of core genes, and the online tool iTOL v6^15^ was used to visualize the tree.

### String test and biofilm formation assay

A string test was done using a standard inoculation loop to gently lift a colony of DY1928 grown on a blood agar plate to detect the hypermucoid phenotype, and the string test was rated as positive if a mucoid string of > 5 mm was observed (Neumann et al., 2022). Biofilm formation assay was conducted according to the methods described in previous studies (Heiden et al., 2020). Bacterial suspensions were diluted to 0.5 Mc (around 10^8^ cfu/ml) in LB, then were diluted 100 times by LB to 10^6^ cfu/ml. 200 μl of the two different concentrations (10^8^ cfu/ml and 10^6^ cfu/ml) of bacterial suspension and sterile LB (negative control) were inoculated into 96-well microplates (Corning Life Sciences, New York, United States) in seven replicates at 37°C for 24 h. Then the medium was removed, and the wells were washed with PBS and fixed with methanol. Staining was performed with 200 μl of 0.1% (w/v) crystal violet solution for 15 min at room temperature. The wells were repeatedly washed with PBS to remove excess stain and dried at 37°C for 1 h. The biofilm formation was quantified by solubilizing the crystal violet stain. Thereafter, the optical density (OD) was read on a multi-detection microplate reader (Bio-Rad Laboratories, Hercules, United States) at 595 nm. Experiments were performed in triplicate and results were presented as mean ± SD. The data were analyzed as described before (Brahma et al., 2019).

The following criteria were used to interpret the results:

ODc ≥ ODs: non biofilm formation.

ODc < ODs ≤ 2*ODc: weak biofilm formation.

2*ODc < ODs ≤ 4*ODc: moderate biofilm formation.

4*ODc < ODs: strong biofilm formation.

[ODc is the mean OD of the negative control; ODs is the mean OD of samples].

### Mouse sepsis model construction and analysis

Three *K. pneumoniae* strains, DY1928, ATCC 700603 (standard control), and K950 (high virulent control carrying *bla*_NDM-1_) (Zheng et al., 2020a) were used to construct the mouse sepsis model. According to a previous study (Jin et al., 2021), female BALB/C mice (seven weeks old) were adaptively housed in the laboratory for seven days before the experiment, and mouse feces were collected on day 7. All of the bacterial strains were cultivated for nine hours (the post-exponential phase) After that, the cells were adjusted to Mc = 0.5 with sterile normal saline (NS), then were diluted 100 times by sterile NS to 10^6^ cfu/ml. 100 μl of the two different concentrations (10^8^ cfu/ml and 10^6^ cfu/ml) of bacterial suspension were injected into mice (n = 4 per strain) *via* the caudal vein. Negative control mice were injected with 100 μl of sterile NS. The physical status of inoculated mice was monitored and recorded daily. Feces were collected from mice in each group on days 1, 3, 5, and 7 after injection. The severity of disease in mice was scored on day 7 according to the Murine Sepsis Score (MSS) system, including seven aspects: appearance, level of consciousness, activity, response to stimulus, eyes, respiratory rate, and respiratory quality. Each of these variables were given a score between 0 and 4 (Shrum et al., 2014; Mai et al., 2018). The highest score was assigned directly when the mouse died. After seven days post-infection, all surviving mice were sacrificed and dissected. Approximately, 10 mg of feces from each mouse was homogenized with 1 ml of sterile NS and then diluted 100 times. 20 μl of each diluted sample was spread on a blood agar plate and incubated overnight at 37°C. Each single colony grown on the plates was picked, purified on MHA plates, and then identified by MALDI-TOF/MS. PCR was then performed on the gram-negative bacteria to detect the presence of *bla*_NDM_*. Bla*_NDM_-positive *K. pneumoniae* strains from DY1928 group were subjected to PFGE, S1-PFGE and Southern blot, and AST, as described below.

### Homology analysis and plasmid characterization

The clonality of DY1928 and the *bla*_NDM_-positive *K. pneumoniae* strains from DY1928 group was confirmed by genomic DNA digestion with XbaI (Sangon, China), followed by pulsed-field gel electrophoresis (PFGE) using the contour-clamped homogenous electric field (CHEF) technique (Shen et al., 2009). Performing the same operation for the DNA marker *Salmonella enterica* serovar Braenderup H9812. The locations of *bla*_NDM-1_ on the plasmids of these strains were validated by S1-PFGE and Southern blot. Briefly, all of the mentioned *K. pneumoniae* strains were embedded in 1% SeaKem Gold gel. They were then digested by S1 nuclease (Takara, Dalian, China) and followed by PFGE. The samples were run on a CHEF-DR II system (Bio-Rad, Munich, Germany) for 16 h at 5 ~ 500 k and 14°C, while the initial and final pulses were 2.16 s and 63.8 s, respectively (Chi et al., 2019). S1-PFGE characterized the plasmid size and number, and the location of *bla*_NDM-1_ was identified by Southern hybridization with a digoxigenin-labelled *bla*_NDM-1_ probe using the DIG-High Prime DNA Labeling and Detection Starter Kit II (Roche, Mannheim, Germany) (Zheng et al., 2016a). Finally, Kit I was used for coloration (Hernández-García et al., 2019).

### Conjugation assay

Conjugal transfer experiments were carried out by using the filter mating method with rifampicin-resistant, plasmid-free strain *Escherichia coli* 600 as the recipient (Hameed et al., 2021), and *K. pneumoniae* strain DY1928 as the donor. Transconjugants were selected on agar medium (OXOID, Hampshire, UK) supplemented with meropenem (2 μg/ml) and rifampicin (200 μg/ml). The transconjugants were further identified by MALDI-TOF/MS, and the presence of *bla*_NDM_ was confirmed by PCR.

### Antimicrobial susceptibility testing

Bacterial strains were cultivated overnight on blood agar plates at 37°C. *E. coli* ATCC 25922 and *K. pneumoniae* ATCC 700603 were used as controls (Zheng et al., 2020b). The minimum inhibitory concentrations (MICs) of meropenem, imipenem, ceftriaxone, cefotaxime, ceftazidime, cefepime, aztreonam, levofloxacin, ciprofloxacin, amikacin, gentamicin, piperacillin/tazobactam, fosfomycin, chloramphenicol, co-trimoxazole, and amoxicillin/clavulanate were determined by the standard agar dilution method. Results were interpreted according to the most recent report from the Clinical and Laboratory Standards Institute (CLSI; CLSI, 2020). The MICs of tigecycline and polymyxin B were examined using broth micro-dilution (BMD) and interpreted by the criterion of the European Committee on Antimicrobial Susceptibility Testing (EUCAST; Eucast, 2020).

### Statistical analysis

For MSS, data were expressed as mean and SD, and differences between groups were analyzed by applying test for homogeneity of variance and one-way analysis of variance (ANOVA) followed by Dunnett’s pairwise comparison for post-hoc analysis. The data was analyzed using Graphpad Prism Version 8.02, and *p* values less than 0.05 were considered statistically significant.

## Results

### Bacterial isolation and identification

Strain DY1928, isolated from the blood culture of a female neonate with sepsis, was identified as *K. pneumoniae.* Clinical reports showed it was resistant to imipenem with a MIC value of 16 μg/ml. PCR showed it carried a *bla*_NDM_ gene.

### Genomic characterization

The genomic characteristics of *K. pneumoniae* DY1928 were summarized in Supplementary Table 1. The genome of DY1928 consists of one circular chromosome (5,339,005 bp, 57.4% GC content) and one plasmid (147,900 bp, 57.4% GC content). The chromosome contains 5,186 coding genes and 114 RNAs. Notably, four ARGs (*oqxA*, *oqxB*, *fosA*, and *bla*_SHV-12_) were encoded on the chromosome. The plasmid (pNDM-1-DY1928) was classified as IncA/C2-type and harbored 194 coding genes, including *bla*_NDM-1_, *bla*_SHV-12_ and *bla*_SHV-160_. MLST analysis classified DY1928 as ST 25. Virulence profiles of DY1928 are shown in Supplementary Table 2, mainly including fimbriae, capsule, iron uptake system and secretion system. Efflux pump related genes *acrA* and *acrB* were also included. Furthermore, it was predicted that strain DY1928 had two CRISPR loci, both located in the chromosome, with 2 spacers in total. The repeat arrays of the sequence were GCTGCGCCTTACCGGGCCTACGA and ACTATGGTTTTTATGGTTTTTATT, respectively. In Supplementary Table 3, a total of 4 intact prophage regions have been identified.

### Comparative genomic analysis of plasmids

**Figure 1:**
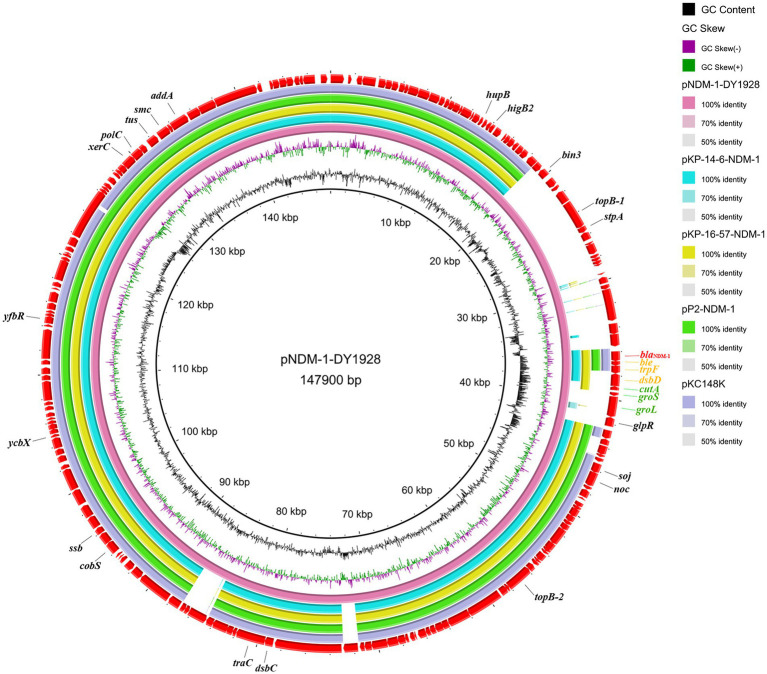
Genome comparison of pNDM-1-DY1928. A comparison of the plasmid pNDM-1-DY1928 sequence with four other plasmids harboring bla_NDM-1_ is shown in the figure. This figure was generated by BRIG.

Four sequenced plasmids with extremely high similarity to pNDM-1-DY1928 were obtained by blasting in GenBank: *K. pneumoniae* plasmids pKP-14-6-NDM-1 (accession number MN175387.1), pKP-16-57-NDM-1 (accession number MZ836809.1) and plasmid pP2-NDM-1 (accession number CP087671.1) from China, and plasmid pKC148K (accession number CP054399.1) from the United States. By comparison, we found that the plasmid pNDM-1-DY1928 from Zhejiang in our study showed great similarity to others in the genomic structure, including the replication, partitioning and transfer systems (Figure 1).

### Genetic environment of *bla*_NDM-1_

**Figure 2:**
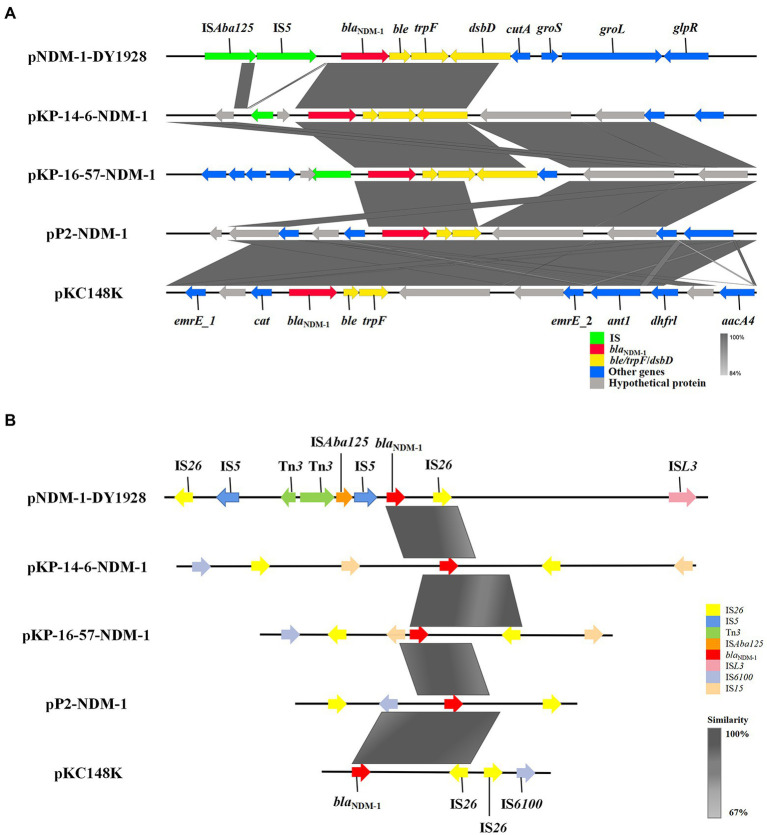
Genetic environment of bla_NDM-1_ on pNDM-1-DY1928 and relevant plasmids: pKP-14-6-NDM-1, pKP-16-57-NDM-1, pP2-NDM-1 and pKC148K. **(A)** Open reading frames (ORFs) are shown as arrows, and are exhibited according to their putative functions. Regions that share a high degree of sequence similarity are indicated by gray. IS elements are colored green, ARGs are red, and conjugal transfer associated genes are yellow. Other genes are colored blue, and assumptive genes that encoded proteins are gray. **(B)** IS elements around the bla_NDM-1_ gene are shown as arrows with corresponding colors indicated in the legend. Regions that share a high degree of sequence similarity are colored as gray.

We found the genetic environment of *bla*_NDM-1_ among the five plasmids to be highly similar. A conservative structure sequence (*bla*_NDM-1_-*ble*-*trpF*-*dsbD*) was found downstream of *bla*_NDM-1_ (Figure 2A). In addition, the IS elements surrounding *bla*_NDM-1_ (Figure 2B) mostly belong to the IS*6* family and show high similarities among the relevant plasmids. It’s worth noting that the plasmid pNDM-1-DY1928 carries the IS*5* transposase, the IS*Aba125* transposase (IS*30* family), the IS*L3* transposase genes, and the Tn*3* transposons. These may be involved in the horizontal transfer of *bla*_NDM-1_.

### Phylogenetic tree analysis

**Figure 3:**
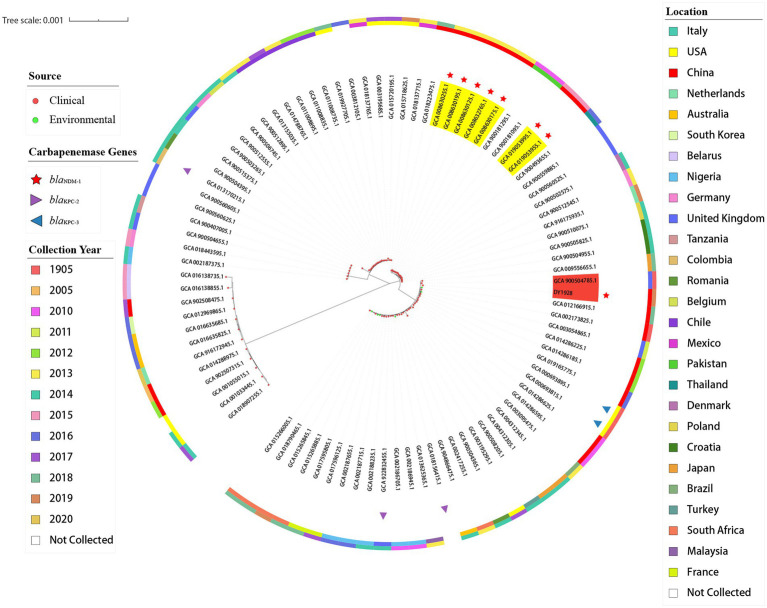
Phylogenetic trees of 91 *K. pneumoniae* strains of ST25. In the figure, the middle part is the maximum likelihood core-gene phylogeny generated by MEGA11. The sources, carbapenemase genes carried, locations, and collection years of all ST25 *K. pneumoniae* strains included here are indicated in the figure.

As shown in Figure 3, among all of the 90 ST25 *K. pneumoniae* strains, strain DY1928 was most closely related to strain EuSCAPE_UK019 (GCA_900504785.1) from the United Kingdom, carrying no carbapenem-resistant genes. In addition, phylogenetic analysis showed that strains P23 (GCA_008630255.1), P35 (GCA_008630195.1), P36 (GCA_008630175.1), P37 (GCA_008632765.1) and P42 (GCA_008630125.1) were intimately related and were all isolated from China in 2013. Also, strains xz032 (GCA_019053955.1) and xz033 (GCA_019053995.1) shared a close phylogenetic relationship, and both were isolated from China in 2015. All seven of the above strains carried *bla*_NDM-1_ and were isolated from the clinic.

### *In vitro* characterization and *in vivo* virulence

**Figure 4:**
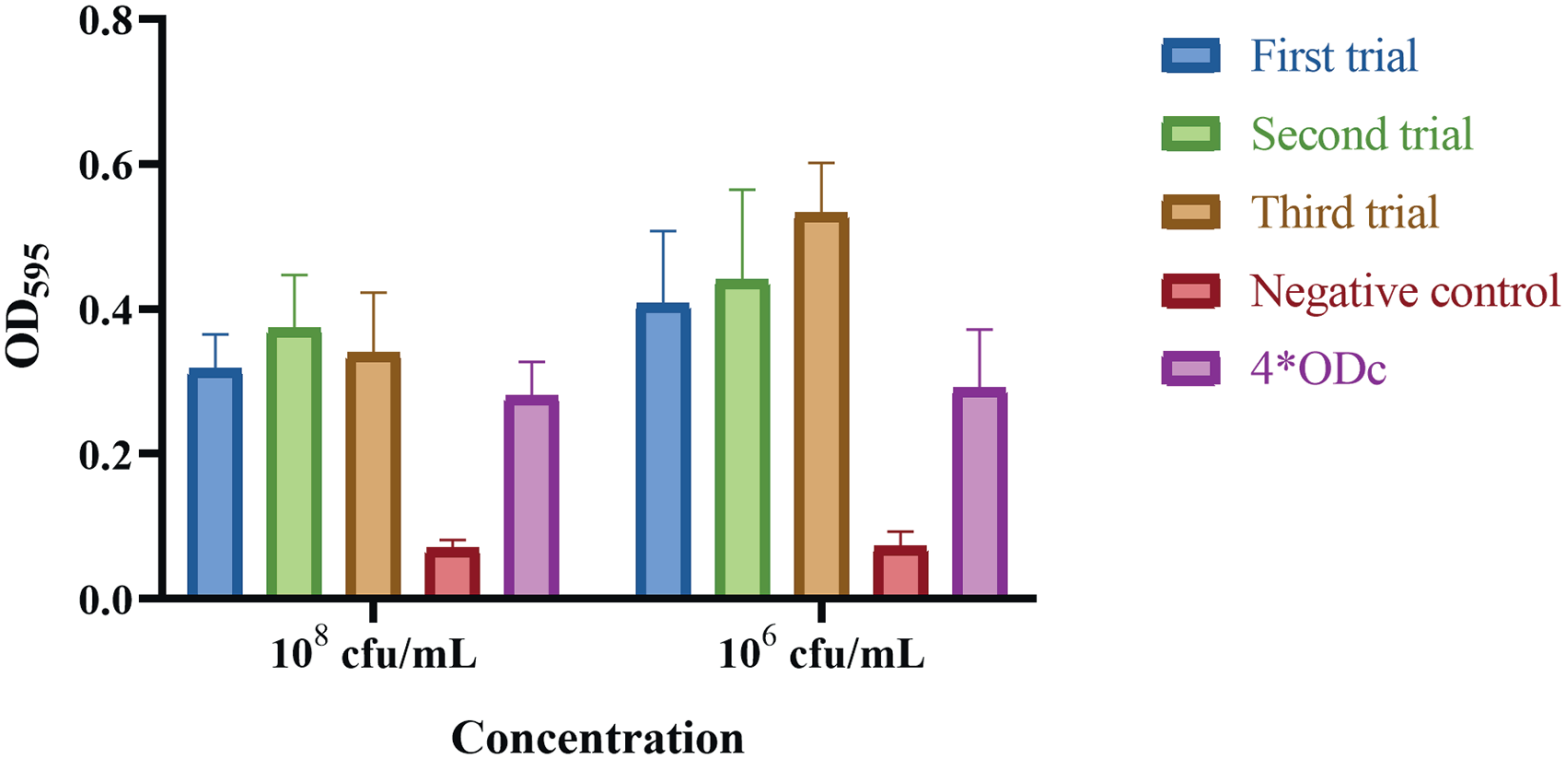
The biofilm biomass of *K. pneumoniae* DY1928. In the figure, ODc is the mean OD of the negative control. Criteria for judgment are: non-biofilm formation, ODc ≥ ODs; weak biofilm formation, ODc < ODs ≤ 2*ODc; moderate biofilm formation, 2*ODc < ODs ≤ 4*ODc; strong biofilm formation, 4*ODc < ODs (ODs is the mean OD of the samples).

Strain DY1928 showed a negative string test with a mucoid string of < 5 mm. The biofilm assay shown in Figure 4 revealed that DY1928 had a strong capacity for biofilm formation, with all mean OD values of trial groups greater than 4*ODc. According to the MSS system, the disease severity of mice in DY1928 group was more severe than in NS and standard control groups, but less severe than in high virulent control group, and the disease severity at 10^8^ cfu/ml was more severe than at 10^6^ cfu/ml (Supplementary Figure 1). All mice except for the K950 group (at 10^6^ cfu/ml and 10^8^ cfu/ml) survived on day seven. We only managed to isolate *bla*_NDM-1_-positive *K. pneumoniae* strains in DY1928 and K950 groups at 10^8^ cfu/ml. In total, 11 *K. pneumoniae* strains were isolated from DY1928 group at 10^8^ cfu/ml and numbered m1-1.1d, m3-2.1d, m4-1.1d, m1-1.3d, m2-1.3d, m4-1.3d, m2-1.5d, m4-1.5d, m1-1.7d, m2-1.7d, m4-1.7d (Note: “m” represents mouse, “d” represents day. e.g., m1-1.1d represents the first strain isolated from the first mouse of DY1928 group on day one).

### Homology analysis and plasmid typing

**Figure 5:**
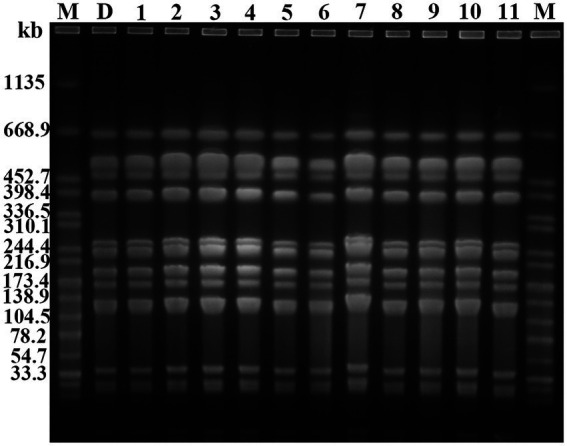
Genome atlas of the target strains by PFGE. “M” represents the marker: *Salmonella enterica* serotype Braenderup H9812, D: DY1928, 1: m1-1.1d, 2: m3-2.1d, 3: m4-1.1d, 4: m1-1.3d, 5: m2-1.3d, 6: m4-1.3d, 7: m2-1.5d, 8: m4-1.5d, 9: m1-1.7d, 10: m2-1.7d, 11: m4-1.7d (“m” represents mouse, “d” represents day. e.g., m1-1.1d represents the first strain isolated from the first mouse of DY1928 group on day one).

**Figure 6:**
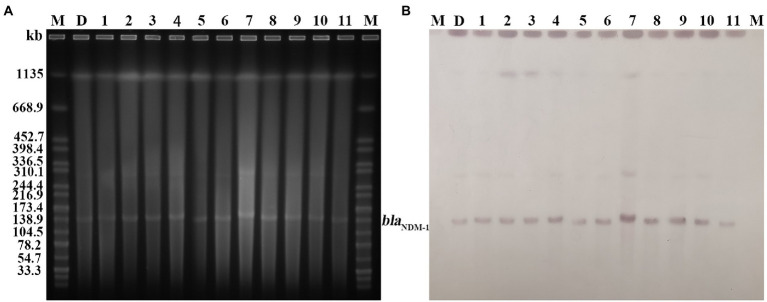
Plasmid profiles of the target strains. Letters and numbers indicate the same contents as in Figure 5. **(A)** Plasmid size determination by S1-PFGE. The bright bands at the top of each lane are the chromosomes of each strain, and the bright bands below are the plasmids. **(B)** Southern blot hybridization with plasmids using a *bla*_NDM_ probe. The position of the blots indicates the position of the plasmids.

PFGE analysis indicated that all 11 *K. pneumoniae* strains isolated from mouse feces of DY1928 group were the same clonal patterns as DY1928, therefore they were all homologous to DY1928 (Figure 5). Further analysis of S1-PFGE and Southern blot showed that the *bla*_NDM-1_ gene was carried by a plasmid with a length of 147.9 kb (Figure 6). Also, the above 11 *K. pneumoniae* strains showed the same plasmid patterns as DY1928 (Figure 6). Therefore, the above results together demonstrated that *K. pneumoniae* DY1928 could enter the intestine from blood in mice without losing the plasmid carrying *bla*_NDM-1._

### Bacterial conjugation

Two transconjugants, DY1928-*E. coli* 600 ① and DY1928-*E. coli* 600 ②, were selected and identified as *E. coli* by MALDI-TOF MS. They were also found to be *bla*_NDM_-positive by PCR (Supplementary Figure 2). Thus, it was demonstrated that the plasmid carrying *bla*_NDM-1_ from the donor DY1928 could transfer into receptor bacteria.

### Antimicrobial susceptibility

Antimicrobial susceptibility analysis (Table 1) showed that strain DY1928 was sensitive to aztreonam, levofloxacin, ciprofloxacin, amikacin, gentamicin, fosfomycin, chloramphenicol, co-trimoxazole, and tigecycline. While displaying intermediate resistance to polymyxin B, it showed resistance to meropenem, imipenem, ceftriaxone, cefotaxime, ceftazidime, cefepime, piperacillin-tazobactam, and amoxicillin-clavulanate. The drug resistance profiles of the two transconjugants were similar to that of DY1928, except for DY1928-*E. coli* 600 ①, which showed intermediate resistance to piperacillin-tazobactam. *E. coli* 600 was sensitive to all of the above drugs. The MICs of 11 *K. pneumoniae* strains isolated from mouse feces of DY1928 group were shown in Table 2. They all showed the same antimicrobial susceptibility as DY1928.

**Table 1:** MICs of DY1928, transconjugants and *E. coli* 600.

Antimicrobials	MIC values (μg/mL)			
	DY1928	DY1928-*E. coli* 600 ①	DY1928-*E. coli* 600 ②	*E. coli* 600
Meropenem	4/R	16/R	8/R	0.06/S
Imipenem	32/R	4/R	4/R	0.5/S
Ceftriaxone	>128/R	128/R	>128/R	0.125/S
Cefotaxime	128/R	128/R	>128/R	0.25/S
Ceftazidime	>128/R	>128/R	>128/R	0.5/S
Cefepime	32/R	16/R	16/R	0.03/S
Aztreonam	0.03/S	0.06/S	0.06/S	0.125/S
Levofloxacin	0.125/S	0.125/S	0.125/S	0.125/S
Ciprofloxacin	0.06/S	0.25/S	0.5/S	0.25/S
Amikacin	4/S	4/S	4/S	4/S
Gentamicin	1/S	0.5/S	0.5/S	1/S
Piperacillintazobactam	>128/R	64/I	>128/R	4/S
Fosfomycin	1/S	0.5/S	0.5/S	0.5/S
Chloramphenicol	8/S	4/S	8/S	8/S
Co-trimoxazole	0.125/S	0.125/S	0.125/S	0.125/S
Amoxicillinclavulanate	>128/R	>128/R	>128/R	4/S
Tigecycline	0.06/S	0.06/S	0.06/S	0.25/S
Polymyxin B	2/I	1/I	1/I	0.5/I

S, sensitive; R, resistant; I, intermediate.

**Table 2:** MICs of 11 *K. pneumoniae* strains isolated from mouse feces of DY1928 group.

Antimicrobials	MIC values (μg/mL)										
	1	2	3	4	5	6	7	8	9	10	11
Meropenem	8/R	4/R	8/R	8/R	8/R	8/R	4/R	4/R	4/R	4/R	4/R
Imipenem	16/R	16/R	16/R	16/R	16/R	16/R	8/R	32/R	16/R	8/R	16/R
Ceftriaxone	128/R	128/R	128/R	128/R	128/R	128/R	128/R	128/R	128/R	128/R	128/R
Cefotaxime	64/R	64/R	64/R	128/R	128/R	64/R	64/R	128/R	64/R	64/R	64/R
Ceftazidime	>128/R	>128/R	>128/R	>128/R	>128/R	>128/R	>128/R	>128/R	>128/R	>128/R	>128/R
Cefepime	32/R	16/R	16/R	32/R	32/R	16/R	16/R	16/R	16/R	16/R	32/R
Aztreonam	0.125/S	0.125/S	0.125/S	0.125/S	0.125/S	0.25/S	0.125/S	0.125/S	0.125/S	0.125/S	0.125/S
Levofloxacin	0.125/S	0.125/S	0.125/S	0.06/S	0.06/S	0.06/S	0.06/S	0.06/S	0.06/S	0.06/S	0.125/S
Ciprofloxacin	0.03/S	0.03/S	0.03/S	0.06/S	0.06/S	0.06/S	0.06/S	0.06/S	0.06/S	0.06/S	0.03/S
Amikacin	4/S	4/S	4/S	2/S	2/S	2/S	2/S	2/S	2/S	2/S	4/S
Gentamicin	2/S	2/S	2/S	1/S	1/S	1/S	1/S	1/S	1/S	1/S	2/S
Piperacillin-	>128/R	>128/R	>128/R	>128/R	>128/R	>128/R	>128/R	>128/R	>128/R	>128/R	>128/R
tazobactam											
Fosfomycin	1/S	1/S	1/S	0.5/S	0.5/S	1/S	1/S	16/S	0.5/S	0.5/S	1/S
Chloramphenicol	8/S	8/S	8/S	8/S	8/S	8/S	8/S	8/S	8/S	8/S	8/S
Co-trimoxazole	0.25/S	0.25/S	0.25/S	0.25/S	0.25/S	0.125/S	0.125/S	0.25/S	0.125/S	0.125/S	0.25/S
Amoxicillin-	64/R	64/R	64/R	64/R	64/R	64/R	64/R	64/R	64/R	64/R	64/R
clavulanate											
Tigecycline	0.25/S	0.25/S	1/S	0.5/S	0.5/S	0.25/S	0.25/S	0.25/S	0.125/S	0.25/S	0.5/S
Polymyxin B	2/I	2/I	2/I	2/I	2/I	2/I	2/I	2/I	2/I	2/I	2/I

S, sensitive; R, resistant; I, intermediate; 1: m1-1.1d, 2: m3-2.1d, 3: m4-1.1d, 4: m1-1.3d, 5: m2-1.3d, 6: m4-1.3d, 7: m2-1.5d, 8: m4-1.5d, 9: m1-1.7d, 10: m2-1.7d, 11: m4-1.7d (“m” represents mouse, “d” represents day. e.g., m1-1.1d represents the first strain isolated from the first mouse of DY1928 group on day one).

## Discussion

In the present study, one NDM-1-producing *K. pneumoniae* strain, DY1928, was isolated from an only 9-day-old female neonate who was diagnosed with an intracranial infection. The blood culture indicated that the neonate was infected with CRKP. The neonate was treated with a combination of penicillin sodium and meropenem for 4 days, followed by 17 days of treatment with meropenem alone. This resulted in an improvement in the neonate’s condition. Following 4 days of treatment with meropenem alone, two consecutive blood cultures became negative. After 30 days in the hospital, the neonate recovered. The increase in antimicrobial resistance among bacteria is making existing antimicrobial agents less effective. There are currently limited treatment options available for neonatal sepsis, particularly for infections caused by CRKP. Meropenem has been shown to be effective in treating NDM-producing *K. pneumoniae* sepsis when used in combination with other agents, such as fosfomycin, or in high doses (Zou et al., 2022). Although DY1928 showed *in vitro* resistance to meropenem with a MIC of 4 μ/ml, this case achieved good clinical efficacy. This may be due to the high dose of meropenem used and combination therapy in clinical practice, or the *in vivo* antimicrobial resistance of *bla*_NDM-1_ may be inhibited. Thus, the combined use of penicillin sodium and meropenem in the present study may provide some reference for the treatment of neonatal sepsis caused by NDM-1-producing *K. pneumoniae* (Jin et al., 2017; Zou et al., 2022).

We speculated that the CRKP might have been transmitted by hospital settings due to the high horizontal transfer capability of *bla*_NDM-1_, and the relatively long hospitalization periods of the patients (Yu et al., 2017). A previous study also indicated that water in incubators and sharing of breast milk were “hotspots” for bacterial transmission in neonatal wards (Zheng et al., 2016b). Besides, underlying disease, low birth weight, low immunity, intrauterine infections and invasive procedures, all of which have been reported as primary risk factors for CRE infection in neonatal wards (Jiao et al., 2015). Additionally, the overuse of carbapenems and cephalosporins is another important risk factor (Candevir Ulu et al., 2015).

There have been numerous reports of NDM-1-producing *K. pneumoniae* of different STs causing infections in humans (Huang et al., 2018). *Bla*_NDM-1_ genotype *K. pneumoniae* is a major cause of neonatal carbapenem-resistant sepsis in China (Ding et al., 2019). To date, there have been no reports of infection caused by NDM-1-producing ST25 *K. pneumoniae* in neonatal units. Previous studies on the spread of CRKP have largely focused on adults, leaving a significant void in neonates (Zhang et al., 2018; Zou et al., 2022). Moreover, several studies have found that CRKP has a multiclonal background and is more genetically diverse among children than adults (Xiao et al., 2017; Zhang et al., 2018). Therefore, it is essential to study the genomic characteristics, phylogenetic, and virulent features of ST25 CRKP isolated from neonates with sepsis.

Previous studies indicated that the *bla*_NDM-1_ gene was predominantly found in *K. pneumoniae* ST25 strains, and CRKP strains were always dominated by lineage ST25 (Zhang et al., 2018, 2020). ST25 appeared to have evolved into multi-drug resistance (MDR; Yao et al., 2015). All ST25-type *K. pneumoniae* strains that carried *bla*_NDM-1_ were found to be isolated from China, according to the phylogenetic tree. As previously described, strains P23, P35, P36, P37, and P42 were isolated from Guangdong in 2013, and strains xz032 and xz033 were isolated from Jiangsu in 2015. These findings may indicate the widespread propagation across time and space of the *bla*_NDM-1_ gene in China. ST25 was proved to be a hypermucoviscous and hypervirulent clone (Cejas et al., 2019). A study found that the ST25 CR-hvKP strains had a clonal distribution in hospitals in China (Li et al., 2019). In the present study, strain DY1928 showed a negative string test. Although bacterial hypermucoviscosity is related to its high virulence, it’s not necessary (Wei et al., 2022). DY1928 also exhibited a strong ability to form biofilms. The antimicrobial resistance of *K. pneumoniae* strains increased significantly when the strains grew as biofilms (Vuotto et al., 2014). This may suggest that such *K. pneumoniae* ST25 strains have the ability to cause clinical challenges. Moreover, DY1928 was found to carry various virulence factors, including type 1 and type 3 fimbriae, as well as the capsule and the LPS. These virulence factors mostly contribute to the ability of *K. pneumoniae* to grow as biofilms (Vuotto et al., 2014). These hypervirulent genes participate in the process of bacterial infection. For example, the *fimH* gene encodes type 1 fimbriae, which plays an important role in urinary tract infections (Vargas et al., 2019). The *mrkA* and *mrkD* genes encode type 3 fimbriae, which promote biofilm formation (Struve et al., 2009; Jure et al., 2021). The *iutA*, *entB* and *iroN* genes encode iron-binding proteins that promote biofilm development (El Fertas-Aissani et al., 2013). Additionally, the simultaneous decreased susceptibility to multiple antimicrobials may be caused by the efflux pump *acrAB*. A report showed that *K. pneumoniae* harboring *bla*_NDM-1_ was the most virulent in the mice sepsis model, and a strong biofilm producer (Fuursted et al., 2012). Therefore, the increasing prevalence of *bla*_NDM-1-_positive ST25 *K. pneumoniae* in various regions of China has become an emerging threat.

By comparing the genetic environment of *bla*_NDM-1_ genes harbored by pNDM-1-DY1928 with that of other four plasmids (pKP-14-6-NDM-1, pKP-16-57-NDM-1, pP2-NDM-1 and pKC148K), a conservative structure sequence (*bla*_NDM-1_-*ble*-*trpF*-*dsbD*) was found downstream of *bla*_NDM-1_, which may be the core structure of horizontal transfer of *bla*_NDM-1_ (Zhang et al., 2020). For pNDM-1-DY1928, it contains a classically conservative region (*bla*_NDM-1_-*ble*-*trpF*-*dsbD*-*cutA*-*groS*-*groL*), which is probably involved in the further dissemination of *bla*_NDM − 1_ (Cai et al., 2019). Various plasmids encoding NDM-1 with highly similar genetic structures have been observed in other studies, such as in *K. pneumoniae* from China (*rmtC*-ΔIS*Aba125*-*bla*_NDM-1_-*ble*-*trpF*-*dsbC-cutA*-*groS*-*groL*; Zheng et al., 2018), *K. pneumoniae* from Australia (*bla*_NDM − 1_-*ble*-*trpF*-*tat*-*dct*-*groS*-*groL*; Wailan et al., 2016), *Enterobacter cloacae* from China (*bla*_NDM − 1_-*ble*-*trpF*-*dsbD*-*cutA1*-*groS*-*groL*; Cai et al., 2019), and *Citrobacter freundii* from China (IS*5*-*bla*_NDM-1_-*trpF*-*dsbC*-*cutA1*-*groE*; Yang et al., 2015). Furthermore, it has been suggested that the *bla*_NDM-1_ gene was derived from *Acinetobacter baumannii* due to the presence of the *ble* gene in close proximity, and *K. pneumoniae* was thought to be a key host in the preservation of *bla*_NDM–1_ (Poirel et al., 2011).

Meanwhile, the IS elements around *bla*_NDM-1_ could help us to reveal the genetic characteristics of this gene. Notably, as our study revealed, *bla*_NDM-1_ in pNDM-1-DY1928 is located between the Tn*3* and IS*26* transposase genes (Figure 2B). It was demonstrated that transposons (Tn*3* and IS*26*) located in the upstream or downstream of the *bla*_NDM-1_ gene were often involved in the horizontal transfer of ARGs (Yong et al., 2009; Nordmann et al., 2011). Thus, the presence of Tn*3* and IS*26* in pNDM-1-DY1928 may also contribute to the transfer of *bla*_NDM-1_. Besides, *bla*_NDM-1_ was usually linked with IS*30* family mobile elements, such as IS*Aba125,* which may relate to the horizontal transfer of *bla*_NDM-1_ from the original host (Zhang et al., 2020). In addition, IS*5* was frequently presented in the genetic context of *bla*_NDM-1_ (Chen et al., 2020; Shin et al., 2021). Furthermore, all *bla*_NDM_ variants shared similar genetic environments, which included the presence of the conserved sequences IS*Aba125*, IS*26*, Tn*3*, and IS*5* (Stokes and Gillings, 2011; Giufrè et al., 2018). These highly conserved structures suggested a common genetic origin among the *bla*_NDM_ variants, and may be related to the horizontal transfer of *bla*_NDM_ (Chakraborty et al., 2021). It was suggested that phages could be a reservoir for spreading ARGs by transduction (Zemmour et al., 2021). In addition to ARGs, the CRISPR-Cas system may confer an adaptive immunity against mobile genetic elements (MGEs) (Makarova et al., 2015). Above all, it was shown that *K. pneumoniae* DY1928 had high dissemination and transfer ability.

Animal models are one of the methods to identify the virulence of *K. pneumoniae* strains (Albarracin et al., 2022). In the mouse experiment, we isolated 11 *K. pneumoniae* strains that harbored *bla*_NDM-1_ homologous to DY1928. DY1928 was found to be able to enter the intestine from the blood in mice without losing the plasmid carrying *bla*_NDM-1,_ and was able to cause mice damage with stronger virulence than the standard control strain. Notably, when mice were dissected, we observed multiple organ damages in DY1928 group, such as overlying blisters and plaques, and splenomegaly. Moreover, the lesions in the group at 10^8^ cfu/ml were more significant than those in the group at 10^6^ cfu/ml. On the one hand, the virulence of the strain was mainly illustrated by whether the strain crossed the intestinal barrier from the mouse’s blood into the intestine. On the other hand, it was illustrated auxiliarily by the MSS score. There have been several studies indicating the high virulence of CRKP strains in mice. Methods such as abdominal injection (Bai et al., 2022), intravenous injection (Tian et al., 2021), nasal drip (Lin et al., 2020), and feeding (Fang et al., 2022) were used. However, to our knowledge, there are few studies on the transfer and colonization of CRKP from blood to the intestine in mice. Furthermore, there were no studies that characterized the virulence of *K. pneumoniae* ST25 strains in animal models except the study of Albarracin et al. (2022). Our findings indicated the high virulence of strain DY1928, together with its resistance to multiple antimicrobial agents. Thus, such strains should be under surveillance and control.

Our study has several limitations. First, due to the lack of information on the specific progress of the case, surveillance and culture samples were not investigated from the NICU environment and flora of neonates, the origin of the NDM-1-producing isolate in our study is still unknown. Second, the sample size in the existing study is small and not deep enough to elucidate the specific metastasis and injury mechanism of CRKP strains in mice. However, further research is being planned.

## Conclusion

In conclusion, we reported the characteristics of an NDM-1-producing ST25 *K. pneumoniae* causing infection in a neonatal unit for the first time. Our findings indicated that this high-risk strain might have become an emerging threat in China, especially in neonatal units. Therefore, rigorous surveillance and strict infection control measures are critical to preventing the transmission of such strains.

## Data availability statement

The datasets presented in this study can be found in online repositories. The names of the repository/repositories and accession number(s) can be found in the article/Supplementary material.

## Ethics statement

The studies involving human participants were reviewed and approved by Clinical Research Ethics Committee of the First Affiliated Hospital, Zhejiang University, School of Medicine. The patients/participants provided their written informed consent to participate in this study. The animal study was reviewed and approved by the Tab of Animal Experimental Ethical Inspection of the First Affiliated Hospital, College of Medicine, Zhejiang University.

## Author contributions

WL, XJ, and BZ conceived of and designed the study and critically revised the manuscript. JZ and HX performed the sampling and analyzed the data. JZ, HX, JL, and TS performed the experiments. JZ completed the first draft of the manuscript. All authors contributed to the article and approved the submitted version.

## Funding

This work was supported by the National Natural Science Foundation of China (grant no. 82072314), Scientific Research Fund Project of Zhejiang Chinese Medical University (2020ZG06), Zhejiang Province Public Welfare Technology Application Research Project (LGN20H280002), and College Students Science and Technology Innovation Project (Xin Miao Talent Project) of Zhejiang Province (2021R410020).

## Conflict of interest

The authors declare that the research was conducted in the absence of any commercial or financial relationships that could be construed as a potential conflict of interest.

## Publisher’s note

All claims expressed in this article are solely those of the authors and do not necessarily represent those of their affiliated organizations, or those of the publisher, the editors and the reviewers. Any product that may be evaluated in this article, or claim that may be made by its manufacturer, is not guaranteed or endorsed by the publisher.

## Footnotes

^1^ https://www.ncbi.nlm.nih.gov/

^2^ https://cge.food.dtu.dk/services/ResFinder/

^3^ https://cge.food.dtu.dk/services/PlasmidFinder/

^4^ https://rast.nmpdr.org/

^5^ https://www-is.biotoul.fr/

^6^ http://www.mgc.ac.cn/VFs/

^7^ http://sourceforge.net/projects/brig

^8^ http://mjsull.github.io/Easyfig/

^9^ https://cge.food.dtu.dk/services/MLST/

^10^ https://crisprcas.i2bc.paris-saclay.fr/CrisprCasFinder/Index

^11^ http://phast.wishartlab.com

^12^ https://github.com/tseemann/prokka

^13^ https://github.com/sanger-pathogens/Roary

^14^ https://megasoftware.net/

^15^ https://itol.embl.de/
