# Supplementary material for: Emergence of a NDM-1-producing ST25 Klebsiella pneumoniae strain causing neonatal sepsis in China
Source: Front Microbiol. 2022 Oct 20;13:980191. doi: 10.3389/fmicb.2022.980191 (PMC9630351; doi:10.3389/fmicb.2022.980191)
Supplement: Supplementary file 1 [file Data_Sheet_1.docx]

1. **Supplementary Figures**

**
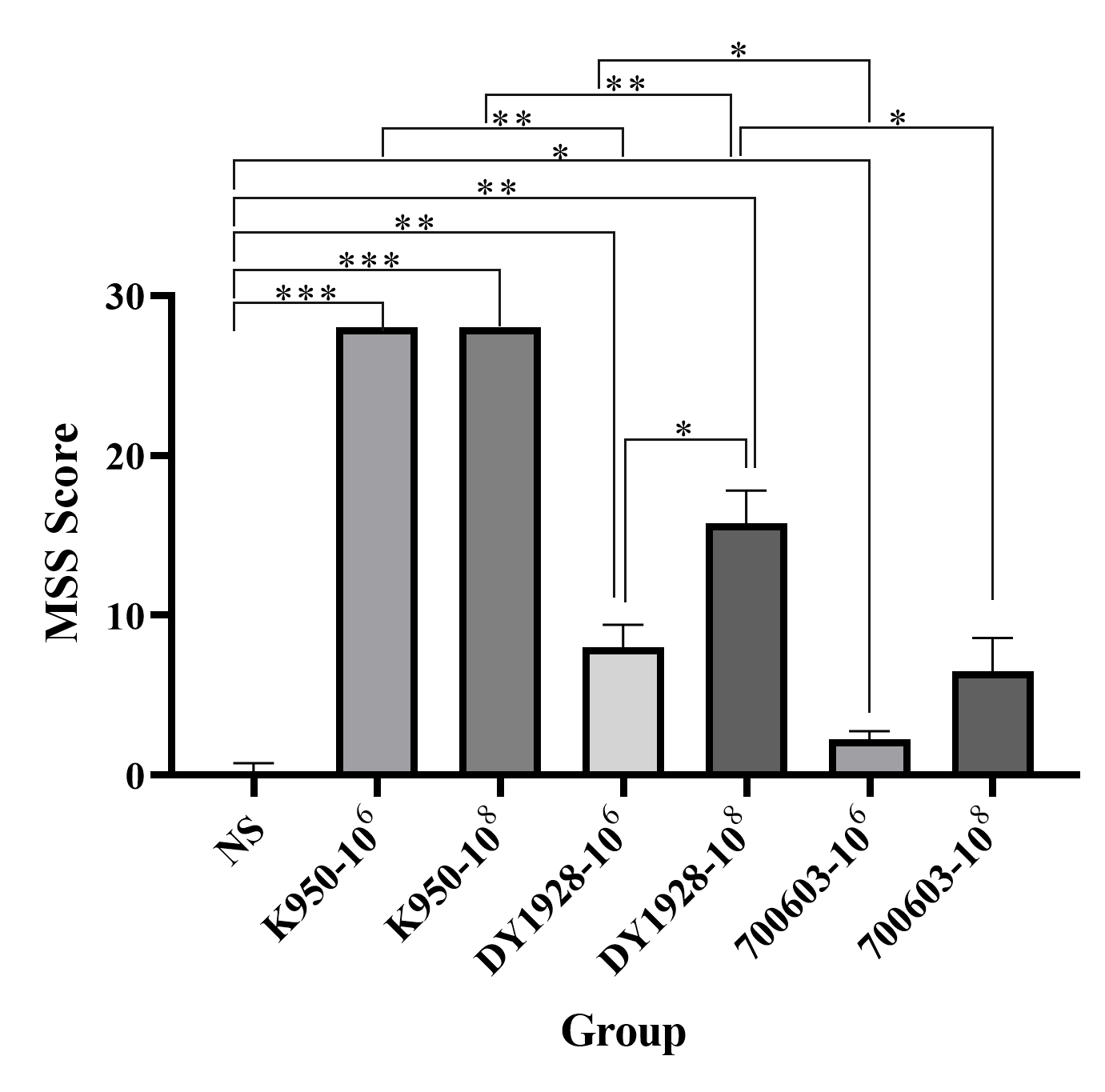
**

**Supplementary Figure 1. Murine Sepsis Score (MSS) on Day 7 after injection.** Results were shown as mean values and SD. **p* < 0.05, ***p* < 0.01, ****p* < 0.001.


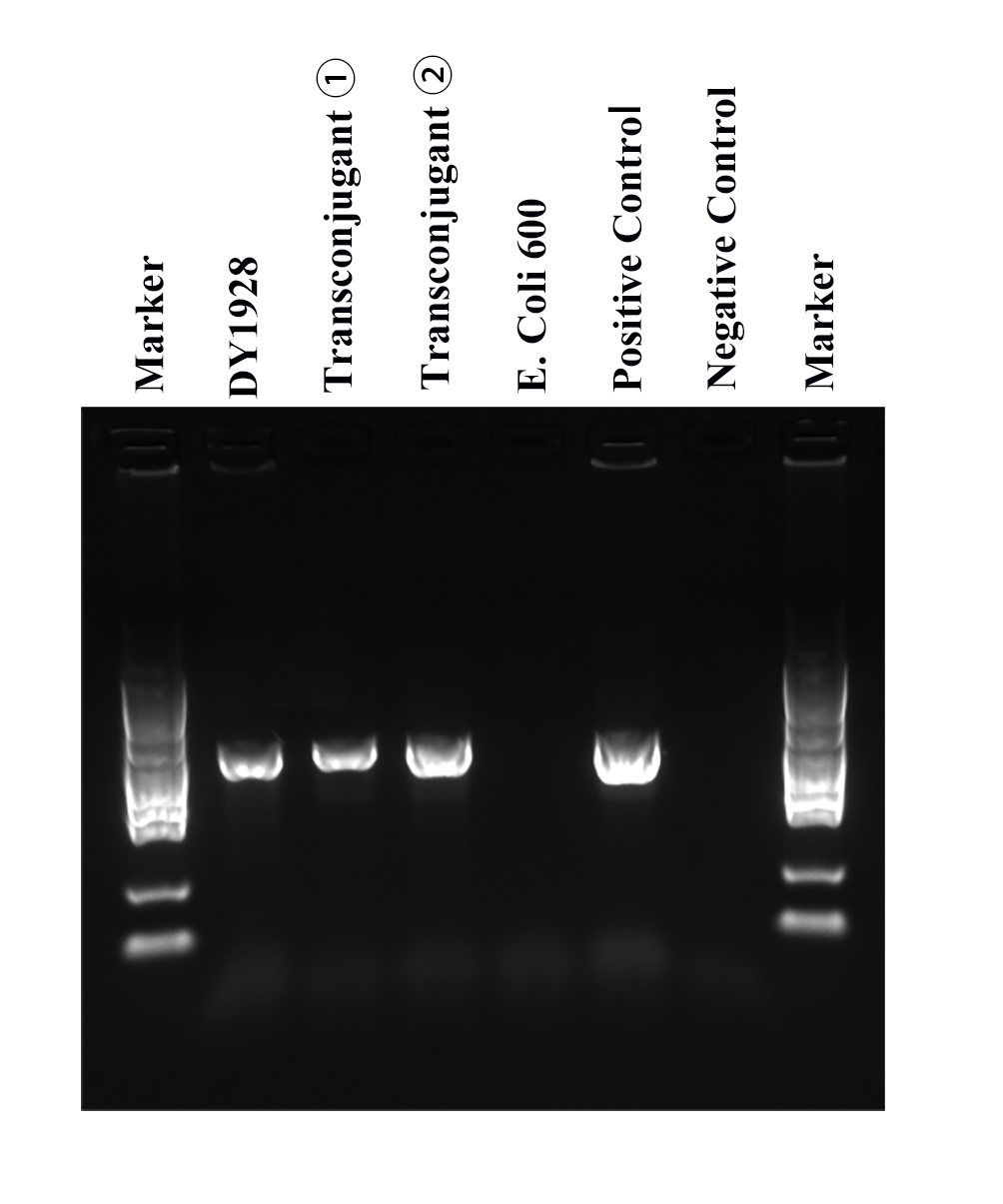


**Supplementary Figure 2.** **Agarose gel electrophoresis of PCR products**. As shown in the figure, marker (DL2000), *K. pneumoniae* DY1928, transconjugants DY1928-*E. coli* 600 ① and ②, *E. coli* 600, positive and negative controls, and marker were ordered from left to right. We see DY1928, the two transconjugants, and positive control have target bands that represent *bla_NDM_* and basically locate in the same location, while *E. coli* 600 and negative control have no purpose band.

1. **Supplementary Tables**

**Supplementary Table 1.** **Genomic features of *K. pneumoniae* DY1928**

| Feature | Chromosome | PNDM-1-DY1928 |
| --- | --- | --- |
| Total number of bases (bp) | 5,339,005 | 147,900 |
| GC content (%) | 57.4 | 51.7 |
| Circular | Yes | Yes |
| Number of coding sequences | 5186 | 194 |
| Number of RNAs | 114 | 0 |
| Plasmid replicon type | - | IncA/C2 |
| Resistance genes | *oqxA, oqxB,*  *fosA, bla_SHV-12_* | *bla_NDM-1_, bla_SHV-12_,*  *bla_SHV-160_* |
| Accession numbers | CP090429 | CP090430 |

**Supplementary Table 2.** **Virulence profiles of *K. pneumoniae* DY1928**

| VF class | Virulence factors | Related genes | *K. pneumoniae* DY1928 (Prediction) |
| --- | --- | --- | --- |
| **Adherence** | Type 3 fimbriae | *mrkA*  *mrkB*  *mrkC*  *mrkD*  *mrkF*  *mrkH*  *mrkI*  *mrkJ* | orf00846  orf00847  orf00848  orf00849  orf00850  orf00853  orf00852  orf00851 |
|  | Type 1 fimbriae | *fimA*  *fimB*  *fimC*  *fimD*  *fimE*  *fimF*  *fimG*  *fimH*  *fimI*  *fimK* | orf00837  orf00841  orf00835  orf00712; orf00834  orf00839  orf00833  orf00832  orf00831  orf00836  orf00830 |
| **Antiphagocytosis** | Capsule | *-* | orf01650; orf01651; orf01652; orf01653; orf01655; orf01658; orf01659; orf01660; orf01662; orf01663; orf01664; orf01665; orf01666; orf04946 |
| **Efflux pump** | acrA  acrB | *acrA*  *acrB* | orf03907  orf03908 |
| **Iron uptake** | Aerobactin | *iucA*  *iucB*  *iucC*  *iucD*  *iutA* | -  -  -  -  orf03128 |
|  | Ent siderophore | *entA*  *entB*  *entC*  *entD*  *entE*  *entF*  *entS*  *fepA*  *fepB*  *fepC*  *fepD*  *fepG*  *fes* | orf03654  orf03655  orf03657  orf03667  orf03656  orf03663  orf03659  orf03666  orf03658  orf03662  orf03660  orf03661  orf03665 |
|  | Salmochelin | *IroB*  *iroC*  *iroD*  *iroE*  *iroN* | -  -  -  orf02578  orf02979 |
|  | Yersiniabactin | *fyuA*  *irp1*  *irp2*  *ybtA*  *ybtE*  *ybtP*  *ybtQ*  *ybtS*  *ybtT*  *ybtU*  *ybtX* | -  -  -  -  -  -  -  -  -  -  - |
| **Nutritional factor** | Allantoin utilization | *allA*  *allB*  *allC*  *allD*  *allR*  *allS* | -  -  -  -  -  - |
| **Regulation** | RcsAB | *rcsA*  *rcsB* | orf01756  orf01526 |
|  | RmpA | *rmpA* | - |
| **Secretion system** | T6SS-I | *-*  *-*  *-*  *clpV/tssH*  *dotU/tssL*  *hcp/tssD*  *icmF/tssM*  *impA/tssA*  *ompA*  *sciN/tssJ*  *tle1*  *tli1*  *tli1*  *tli1*  *tssF*  *tssG*  *vasE/tssK*  *vgrG/tssI*  *vipA/tssB*  *vipB/tssC* | orf02886  orf02876  orf02875  orf02888  orf02891  orf02889  orf02874  -  orf02890  orf02870  -  orf02878; orf02879; orf02880; orf02881; orf02882; orf02883; orf02884; orf02885  orf02872  orf02871  orf02892  orf02887  orf02894  orf02893 |
|  | T6SS-II | *clpV*  *dotU*  *icmF*  *impF*  *impH*  *impJ*  *ompA*  *sciN*  *vasA/impG*  *vgrG* | orf01208  -  -  -  -  -  -  -  -  - |
|  | T6SS-III | *-*  *-*  *-*  *-*  *-*  *-*  *-*  *dotU*  *icmF*  *impA*  *impF*  *impG*  *impH*  *impJ*  *lysM*  *ompA*  *sciN*  *vgrG* | -  orf01918  orf01920  -  orf01923  orf01930  orf01931  orf01916  orf01924  orf01929  orf01928  orf01925  orf01926  orf01915  -  orf01917  orf01927  orf01919 |
| **Serum resistance** | LPS rfb locus | *-* | orf01668; orf01669; orf01670; orf01671; orf01672; orf01673; orf01674 |

**Supplementary Table 3.** **Prophage regions of *K. pneumoniae* DY1928**

| Region | 1 | 2 | 3 | 4 |
| --- | --- | --- | --- | --- |
| Region length (Kb) | 39.9 | 59.6 | 32.6 | 45.6 |
| Completeness | intact | intact | intact | intact |
| Score | 150 | 130 | 150 | 120 |
| CDS | 47 | 61 | 39 | 67 |
| Region position | 1426498-  1466428 | 2134725-  2194384 | 3473591-  3506190 | 3954890-  4000521 |
| Phage species number | 23 | 29 | 5 | 32 |
| GC content (%) | 49.58 | 52.41 | 51.73 | 52.49 |
